# Role of Multiparametric Ultrasound in Predicting the IDH Mutation in Gliomas: Insights from Intraoperative B-Mode, SWE, and SMI Modalities

**DOI:** 10.3390/jcm14176264

**Published:** 2025-09-05

**Authors:** Siman Cai, Hao Xing, Yuekun Wang, Yu Wang, Wenbin Ma, Yuxin Jiang, Jianchu Li, Hongyan Wang

**Affiliations:** 1 Department of Medical Ultrasound, Peking Union Medical College Hospital, Chinese Academy of Medical Sciences and Peking Union Medical College, Beijing 100730, China; caisiman94@126.com (S.C.);; 2 Department of Neurosurgery, Peking Union Medical College Hospital, Chinese Academy of Medical Sciences and Peking Union Medical College, Beijing 100730, China

**Keywords:** glioma, IDH1, intraoperative ultrasound, molecular markers

## Abstract

**Objectives**: To investigate the correlation between intraoperative conventional ultrasound, SWE, and SMI ultrasound manifestations of glioma and the expression of immunohistochemical markers. **Methods**: Patients with single superficial supratentorial glioma scheduled for brain tumor resection in our neurosurgery department from October 2020 to October 2022 were prospectively included. High-grade glioma (HGG) and low-grade glioma (LGG) were classified by pathological histological grading, and the differences in conventional ultrasound, SWE Young’s modulus, and SMI intratumoral and peritumoral blood flow architecture between HGG and LGG were analyzed, and the SWE diagnostic cut-off value was calculated by the Youdon index. Logistic regression models were used to analyze the independent predictive ultrasound signs associated with the diagnosis of HGG. HGG and LGG were classified by pathological histological grading. IDH1 expression was measured by immunohistochemical methods to analyze the correlation between IDH1 expression in glioma and clinical and ultrasound characteristics. **Results**: Forty-eight patients with glioma admitted to our hospital from October 2020 to October 2022 were included in this study, including 30 (62.5%) with HGG and 18 (37.5%) with LGG. For conventional ultrasound, HGG was often associated with severe peritumoral edema compared with LGG (*p* = 0.048). The sensitivity of HGG was 88.9%, the specificity was 86.7%, and the AUC was 0.855 (95% confidence interval: 0.741–0.968, *p* = 0.001) using Young’s mode 13.90 kPa as the threshold. Logistic analysis showed that SWE Young’s modulus values, and peritumoral and intratumoral SMI blood flow structures, were associated with the diagnosis of HGG. Among the 48 gliomas, 22 (45.8%) were IDH1-positive and 26 (54.2%) were IDH1-negative, with no statistical difference in age between the two groups and a statistical difference in histological grading (*p* < 0.05). There was a statistical difference between IDH1 mutant and wild type in terms of peritumoral edema and SMI intratumoral and peritumoral tissue vascular architecture. Logistic regression models showed that intratumoral and peritumoral tissue SMI vascular architecture was a valid predictor of IDH1 positivity, with a classification accuracy of 81.3%, sensitivity of 90.9%, and specificity of 73.1%. Further group analysis of mutant Young’s modulus values in LGG were higher than wild-type Young’s modulus values (*p* = 0.031). **Conclusions**: Peritumoral and intratumoral tissue SMI vascular architecture was a valid predictor of IDH1 positivity. Based on intraoperative ultrasound multimodality images, we can preoperatively determine the expression of molecular markers of lesions, which is of clinical significance for optimizing surgical strategies and predicting prognosis.

## 1. Introduction

Glioma is the most common primary tumor of the central nervous system, exhibiting considerable heterogeneity and constituting approximately 25% to 28% of all primary brain tumors and 75% to 80% of malignant brain tumors [1,2].

The World Health Organization classifies gliomas based on their pathological tissue characteristics into low-grade gliomas (LGGs) and high-grade gliomas (HGGs). Gliomas of different grades have distinct clinical treatment strategies, and there are significant differences in prognosis [1,3]. Moreover, even among gliomas of the same histologic type and grade, molecular subtypes can lead to substantial variability in treatment response and patient outcomes. Thus, precise classification incorporating molecular markers is essential for guiding clinical decision-making and predicting prognosis. The fifth edition of the World Health Organization (WHO) classification of central nervous system (CNS) tumors (WHO CNS5) in 2021 places greater emphasis on the central role of molecular characteristics in the diagnosis, classification, and grading of CNS tumors compared to the fourth edition in 2016, utilizing gene mutations, chromosomal arm deletions, and molecular variations to determine tumor characteristics [4]. A number of molecular markers have been identified as critical determinants of tumor biology and behavior. Among them, isocitrate dehydrogenase 1 (IDH1) is one of the most extensively studied molecular markers in glioma. Preoperative prediction of IDH1 mutation status based on imaging characteristics is valuable for guiding intraoperative decision-making and predicting patient prognosis [5,6,7].

In 2008, Parsons et al. reported that 12% of glioblastoma patients have IDH1 mutations, which correlated with extended progression-free survival (PFS) and overall survival (OS) [8,9,10]. These mutations most commonly occur at codon 132 of exon 4, where a point mutation leads to the substitution of arginine by histidine. IDH1 wild-type gliomas exhibit greater invasiveness compared to IDH1 mutant gliomas and represent a significant and reasonably independent prognostic indicator. IDH1 mutant gliomas are more predisposed to benefit from gross total resection (GTR). Multiple studies have demonstrated that tumor enhancement on magnetic resonance imaging (MRI) is more prevalent in IDH1 wild-type gliomas compared to IDH1 mutant counterparts, with the degree of enhancement correlating with a relatively poorer prognosis [11,12,13]. In the study by Song et al. [14], IDH1-mutated gliomas on contrast-enhanced MRI were characterized by well-defined margins and mild enhancement. Their regression model achieved a diagnostic sensitivity of 70.70% and a specificity of 80.40% in predicting IDH1 mutation status. Specchia et al. studied the correlation between 5-aminolevulinic acid (5-ALA) and the metabolic activity of glioblastoma multiforme (GBM) cells, and they found that the metabolic activity was more active in IDH wild-type tumors, while the metabolic activity of IDH-mutant tumors was lower [15]. Many research articles have reported the value of using artificial intelligence based on MRI in predicting IDH1 mutations, and the results showed that the most important feature for predicting IDH mutation status was no enhancement or minimal enhancement [16]. In the field of ultrasound, Yu et al. [17] discovered a correlation among the grayscale ultrasonography tumor border, peritumoral edema, and IDH1 expression.

Shear wave elastography (SWE) provides real-time visualization of tissue elasticity using color-coded mapping based on shear wave propagation speed, along with quantitative assessment of Young’s modulus to evaluate local stiffness [18,19]. SWE has been widely applied to evaluate tissue hardness across multiple organs, including the liver, breast, thyroid, prostate, and tendons [18,20,21,22]. Related studies have found that elastography can furnish information regarding tumor stiffness and assist neurosurgeons in ascertaining the characteristics of malignancies [23,24]. Superb microvascular imaging (SMI) utilizes an adaptive algorithm to mitigate clutter produced by tissue motion, enabling the visualization of micro-vessels and low-velocity blood flow signals without the use of a contrast agent, achieving diagnostic efficacy equivalent to contrast-enhanced ultrasound (CEUS) [25]. SMI has been extensively employed in the diagnosis and assessment of tumors, inflammation, and other disorders, significantly improving blood flow assessment and diagnostic accuracy for focal lesions [26,27,28,29].

Preoperative prediction of molecular subtypes in gliomas facilitates the assessment of long-term patient prognosis, thereby enabling neurosurgeons to select appropriate treatment strategies and optimize surgical planning. Currently, there is a lack of studies investigating the association between SWE and SMI features and IDH1 mutation status in gliomas. This study aims to evaluate the predictive value of multimodal ultrasonography indicators for molecular subtyping in gliomas.

## 2. Materials and Methods

The Ethics Committee of Peking Union Medical College Hospital sanctioned this prospective investigation. The advantages and hazards of this study were thoroughly elucidated to the subjects. All patients comprehended and consented to the examination procedure and executed formal informed consent forms. The research was executed in accordance with the principles outlined in the Declaration of Helsinki [30]. Patients with solitary supratentorial superficial glioma, scheduled for brain tumor removal, were prospectively enrolled in the Department of Neurosurgery at our institution from October 2020 to October 2022. Inclusion criteria: 1. Preoperative radiotherapy and chemotherapy were not administered; 2. complete imaging data were accessible, with MRI conducted prior to and within 72 h post-operation, and the lesion assessed via grayscale ultrasound, SWE, and SMI during the procedure; 3. absence of any prior cranial trauma, cranial surgery, or intracranial infection; 4. patients provided informed consent prior to the operation. Exclusion criteria: 1. other pathological forms of cerebral malignancies, including metastases, meningiomas, and lymphomas, were excluded; 2. cases with restricted bone windows that hindered the assessment of lesion borders were eliminated.

### 2.1. Ultrasound Examination

The ultrasound examination employed the AplioTMi900 device (Canon Medical Systems; Tokyo, Japan), utilizing the i6CX1 convex array probe (GE Healthcare, Chicago, IL, USA), which is suitable for investigating brain tumors at varying depths and doing SWE and SMI assessments. The ultrasound assessment of each patient was conducted collaboratively by an ultrasound physician and a minimum of one neurosurgeon. The patient had an ultrasonography examination subsequent to craniotomy and before the dural incision. The probe was sterilized and subsequently coated with a coupling agent on its surface. The probe was encased in a sterile plastic sheath under aseptic circumstances, and the ultrasound probe was positioned vertically on the dural surface for the ultrasonic assessment.

### 2.2. B-Mode Ultrasonography

The ultrasonic probe was positioned vertically on the dura mater with little pressure, and the laterally moved probe was employed to examine the intratumoral and peritumoral structures in B-mode to identify significant anatomical features. The tumor’s location was documented, and its diameter was measured at the largest cross-sectional area. The tumor size was categorized as ≤5 cm and >5 cm based on the maximal diameter. Tumor depth was categorized as ≤2 cm and >2 cm based on the minimum distance from the tumor to the cerebral cortex. The morphology was categorized into regular and irregular forms. The borders were categorized into distinct and ambiguous. Peritumoral edema was categorized into three groups: none, ≤2 cm, and >2 cm, based on the extent of the most pronounced edema. Cystic degeneration and calcification were categorized as either present or absent.

### 2.3. Shear Wave Elastography (SWE)

The tumors were examined in grayscale mode, yielding clear and steady pictures before transitioning to SWE mode. The area of interest was determined based on tumor size, with the tissue within 2 cm of the tumor margin designated as the peritumoral region. The area of focus encompassed the whole lesion and a minimum of 2 cm of surrounding brain tissue. The propagation map represents the isochronous arrival curve of the shear wave at a specified time interval, facilitating the assessment of tissue hardness properties and shear wave propagation. It serves as a reliability index for SWE data collecting, enhancing the precision and consistency of the measurements. The SWE mode was initiated to show the color picture. When the color picture occupied over 80% of the region of interest, the image was captured, and the Q-box was initiated to measure the Young’s modulus of the brain tissue inside the specified area, with the Q-box diameter set to 10 mm. In instances of indeterminate tumor pathology diagnostic findings, sonographers objectively assessed the elasticity values of both intratumoral and peritumoral tissues. The region where the intratumoral and peritumoral tissues aligned horizontally and the propagation map exhibited parallel rippling was designated for measurement. The final Young’s modulus was determined by calculating the mean value of six measurements in the tumor and peritumoral tissues.

### 2.4. Superb Microvascular Imaging (SMI)

SMI functions in two modalities: color SMI and monochrome SMI. The monochrome mode enhances the visibility of the vascular structure by removing the background signal and concentrating on the vessel signal, whereas the color mode presents both the blood flow signal and the grayscale picture. The tumor was initially examined in traditional grayscale mode, then upon achieving a steady and clear picture, the mode was changed to SMI. The gain was calibrated until artifacts were eliminated and the micro-vessels were distinctly seen. The lesions were found in both monochrome and color modes. The dynamic picture was captured by the laterally moving probe at a constant velocity, with a duration of 10–15 s. Two doctors examined SMI dynamic pictures offline; one possessed five years of ultrasound expertise, while the other had four years, with both having over three years of SMI experience. Both of the doctors were blinded to the tumor pathological results, the status of IDH1, the histological grade, and all other patient data. According to the literature report (17) and the summary and analysis in the research process, the vascular architecture of the intratumoral and peritumoral tissues was divided into the following types:

The vascular architecture in the tumor: 1. distorted expansion; 2. straight or branching; 3. no blood vessels.

The vascular architecture around the tumor: 1. twisted surround; 2. straight penetration; 3. normal cerebral vessels.

In cases when the two sonographers’ conclusions on vascular architecture patterns were discordant, a consensus on the vascular architecture evaluation was achieved through discussion.

### 2.5. Immunohistochemical Method

IDH1 mutations: The tumors were preserved in 10% formalin, embedded in paraffin, and sectioned into 10 μm thick slices from which genomic DNA was isolated. The whole coding sequence of exon 4 and codon 132 of the IDH1 gene was amplified using overlapping polymerase chain reaction. They were categorized as “mutant (+)” and “wild type (−)” based on the presence or absence of expression for analysis.

### 2.6. Statistical Methods

Statistical analyses were conducted utilizing SPSS R26.0.0.0 software (IBM Corporation, Armonk, NY, USA). The Shapiro–Wilk test assessed the normality of continuous variables; regularly distributed data were shown as mean ± standard deviation, whereas non-normally distributed data were presented as median and interquartile range. The independent sample *t*-test or Mann–Whitney U test was employed to compare measurement data, the chi-square test was utilized for count data, and Fisher’s exact test was applied when group data were fewer than 5. A binary logistic regression model was utilized for multivariate analysis, and the Hosmer test was employed to assess the goodness of fit of the model. All statistical results were significant at *p* < 0.05.

## 3. Results

This study comprised 48 glioma patients hospitalized in our hospital between October 2020 and October 2022. Of the total, 20 were female (41.7%) and 28 were male (58.3%). Of them, 30 patients (62.5%) had histological grade HGG. The pathological categories comprised the following: glioblastoma, 19 cases (30.6%); anaplastic astrocytoma, 5 cases (10.4%); anaplastic oligodendroglioma, 4 cases (8.3%); gliosarcoma, 1 case (2.1%); and pleomorphic glioblastoma, 1 case (2.1%). A total of 18 patients (37.5%) had histological grade low-grade glioma (LGG). The pathological classifications comprised the following: diffuse astrocytoma, 8 cases (16.7%); oligodendroglioma, 8 cases (16.7%); and ganglioglioma, 2 cases (4.2%). A statistically significant age difference was observed between HGG patients (55.6 ± 14.6 years) and LGG patients (43.6 ± 12.4 years) (*p* < 0.05). No statistically significant difference was seen in gender and tumor site between HGG and LGG patients (Table 1).

The grayscale ultrasonography characteristics of gliomas exhibit significant heterogeneity. No statistical significance exists for shape, border, depth, tumor size, cystic alterations, and calcification between high-grade gliomas (HGGs) and low-grade gliomas (LGGs). Nonetheless, a statistically significant disparity exists in the extent of edema. In the cohort of HGG, 13 patients (43%) exhibited edema within a radius of ≤2 cm surrounding the tumor, whereas 13 cases (43%) presented with edema exceeding 2 cm. LGG had a higher frequency of absence of edema, with a total of 8 instances (8/18, 44.4%) (Table 2).

### 3.1. Results of Intraoperative Superb Microvascular Imaging

During intraoperative SMI, notable changes in blood flow architecture were seen in the tumor and peritumoral tissues between HGG and LGG (*p* < 0.05) (Table 3). The vascular architecture of tumor tissue in high-grade gliomas (HGGs) was predominantly distorted and dilated (18/30, 60.0%), but in low-grade gliomas (LGGs), it was primarily linear or branched (13/18, 72.2%). The blood flow architecture of peritumoral tissue in high-grade gliomas (HGGs) was predominantly convoluted (20/30, 66.7%), but in low-grade gliomas (LGGs), it was less convoluted (2/18, 11.1%) and primarily exhibited straight penetration (9/18, 50.0%) and a typical cerebral vascular configuration (7/18, 38.9%). This study employed Cohen’s kappa coefficient to evaluate the consistency of the two physicians’ assessments of SMI blood flow architecture. The findings indicated that the Cohen’s kappa coefficient for the evaluations of the two physicians was 0.718, with a 95% confidence interval of 0.542–0.894 and a *p*-value of less than 0.001, demonstrating good consistency.

### 3.2. Results of Intraoperative Shear Wave Elastography

The Young’s modulus values for LGG and HGG were 23.4 ± 11.6 kPa and 12.1 ± 13.7 kPa, respectively, with a statistically significant difference (*p* = 0.005). The Young’s modulus of LGG and HGG was 13.2 ± 4.6 kPa and 10.4 ± 3.6 kPa, respectively, with a statistically significant difference (*p* = 0.026). In LGG, a notable disparity in Young’s modulus was seen between the intratumoral and peritumoral tissues (*p* = 0.001). In HGG, the elasticity values between intratumoral and peritumoral tissues exhibited no statistically significant difference (*p* = 0.524) (Table 4). The Young’s modulus of intratumoral tissue was established at 13.90 kPa as the threshold, yielding a sensitivity of 88.9%, specificity of 86.7%, and an AUC of 0.855 (95% confidence interval: 0.741–0.968, *p* = 0.001) for the diagnosis of HGG.

### 3.3. Correlation Between IDH1 Expression and Clinical Features in Gliomas

Of the 48 gliomas, 22 cases (45.8%) were IDH1 mutant, while 26 cases (54.2%) were IDH1 wild type. No substantial variation in patient age was seen between the two groups (Table 3). Within the IDH1 mutation cohort, nine cases (18.8%) had high-grade glioma (HGG) lesions, comprising seven cases (14.6%) of glioblastoma, one case (2.1%) of anaplastic astrocytoma, and one case (2.1%) of anaplastic oligodendroglioma. Thirteen instances (27.1%) of low-grade glioma (LGG) lesions were identified, including six cases (12.5%) of diffuse astrocytoma and seven cases (14.6%) of oligodendroglioma. In the IDH1 wild-type cohort, 21 cases (43.8%) had high-grade glioma (HGG) lesions, comprising 12 cases (25.0%) of glioblastoma, 4 cases (8.3%) of anaplastic astrocytoma, 3 cases (6.3%) of anaplastic oligodendroglioma, 1 case (2.1%) of gliosarcoma, and 1 case (2.1%) of glioblastoma multiforme. Five instances (10.4%) of low-grade glioma (LGG) lesions were identified, including two cases (4.2%) of diffuse astrocytoma, two cases (4.2%) of ganglioglioma, and one case (2.1%) of oligodendroglioma. There were 13 instances of LGG and 9 instances of GGG in the IDH1 mutant type. In IDH1 wild type, there were five instances of LGG and twenty-one instances of HG, which was statistically significant (*p* = 0.004 *).

### 3.4. Correlation Between IDH1 Expression and Ultrasound Features in Gliomas

Ultrasound findings exhibited statistically significant differences between IDH1 mutant and wild type for peritumoral edema, blood flow architecture of SMI peritumoral tissue, and blood flow architecture of SMI tumor tissue. In contrast to IDH1 wild type, IDH1 mutant often exhibited an absence of edema. In comparison to the IDH1 mutant type, the vascular architecture of the peritumoral tissue in IDH1 wild-type SMI was frequently deformed and encircled, whereas the vascular architecture of the intratumoral tissue in SMI was commonly distorted and enlarged (Table 5).

A binary logistic regression model was employed to assess the diagnostic efficiency of peritumoral edema and SMI blood flow architecture in both peritumoral and adjacent tissues for IDH1 expression. No significant difference was seen between peritumoral edema and IDH1 mutation, although SMI blood flow architecture in peritumoral and adjacent tissues well predicted IDH1 mutation (Table 6). The logistic model demonstrated statistical significance (χ^2^ = 23.641, *p* < 0.001). The model achieved a classification accuracy of 81.3%, a sensitivity of 90.9%, a specificity of 73.1%, a positive predictive value of 74.1%, and a negative predictive value of 90.5%. The likelihood of an IDH1 mutation was 11.8% when the SMI blood flow architecture of the tumor tissue was distorted and enlarged, compared to a straight or branching structure with no blood supply. The likelihood of an IDH1 mutation was 12.3% when the SMI blood flow structure of the peritumoral tissue was distorted and encircled, compared to the straight penetrating and normal cerebral vascular morphology.

**Figure 1:**
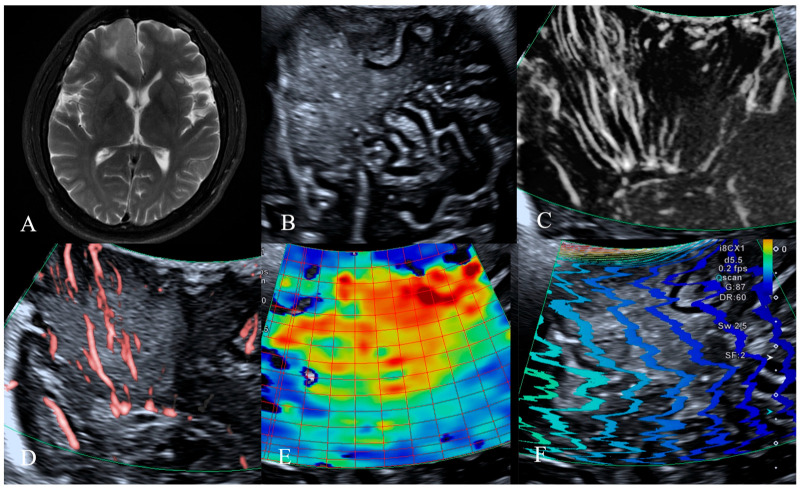
MRI, B-mode, SMI and SWE of LGG in a 46-year-old man. The postoperative pathology was diffuse infiltrating astrocytoma, IDH mutant type. (**A**) Preoperative magnetic resonance imaging T2-weighted images revealed a patchy abnormal signal in the frontal lobe, with mild perilesional edema observed surrounding the lesion. (**B**) B-mode ultrasonography revealed an irregularly shaped, well-defined hypoechoic mass without significant peritumoral edema, internal cystic changes, or calcification. (**C**,**D**) SMI mode, (**C**) is mSMI mode, (**D**) is cSMI mode. The vascular architecture in tumor parenchyma is straight, and the vascular architecture around the tumor is straight penetration. (**E**,**F**) SWE mode. (**E**) Red areas indicate higher stiffness, with the intratumoral region exhibiting significantly increased stiffness compared to the surrounding peritumoral tissue. The mean Young‘s modulus value obtained from repeated measurements of tumor tissue was 54.4 kPa. (**F**) The shear wave propagation map demonstrates that shear waves propagate smoothly within the tumor, with high reliability of data acquisition.

**Figure 2:**
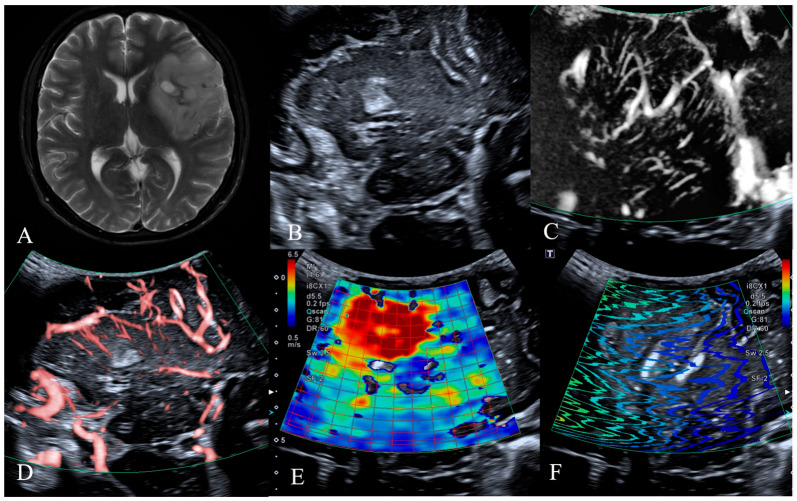
MRI, B-mode, SMI and SWE of LGG in a 54-year-old man. The postoperative pathology was oligodendroglioma, IDH mutant type. (**A**) Preoperative T2-weighted magnetic resonance imaging revealed a large irregular mass in the right fronto-temporo-insular region, exhibiting heterogeneous internal signal intensity with small areas of calcification identified. (**B**) B-mode ultrasonography revealed an irregularly shaped, ill-defined hypoechoic mass without significant peritumoral edema, with internal calcification identified. (**C**,**D**) SMI mode, (**C**) is mSMI mode, (**D**) is cSMI mode. The vascular architecture in tumor parenchyma is straight and branching, and the vascular architecture around the tumor is normal cerebral vessels. (**E**,**F**) SWE mode. (**E**) Red areas indicate higher stiffness, with the intratumoral region exhibiting significantly increased stiffness compared to the surrounding peritumoral tissue. The mean Young‘s modulus value obtained from repeated measurements of tumor tissue was 35.8 kPa. (**F**) The shear wave propagation map demonstrates that shear waves propagate smoothly within the tumor, with high reliability of data acquisition.

The patients were categorized into groups based on glioma grade to further examine the link between the ultrasonography characteristics of LGG and HGG and IDH1 expression. Of the 18 LGG instances, 13 were of the mutant type and 5 were of the wild type. Among all ultrasonography results, only the elasticity value exhibited a statistically significant difference, with the Young’s modulus of the mutant type measuring 27.0 ± 11.3, surpassing that of the wild type at 14.2 ± 6.5 (*p* = 0.031) (Table 7) (Figure 1 and Figure 2). We noticed that in LGG, a twisted and encircled SMI peritumoral blood flow architecture was present only in wild-type IDH1 gliomas, whereas a twisted and enlarged SMI intratumoral vascular architecture was also seen solely in wild-type IDH1 gliomas. In HGG, no significant statistical difference in ultrasonography results was seen between the two groups.

## 4. Discussion

Intraoperative prediction of IDH1 mutation status is crucial for evaluating glioma differentiation, predicting biological behavior and prognosis, and optimizing surgical strategy. However, few studies have focused on predicting molecular markers of brain tumors using intraoperative ultrasonographic features. This study investigated the relationship between grayscale ultrasonography, SWE, SMI characteristics, and IDH1 expression.

IDH1 is a metabolic enzyme involved in the tricarboxylic acid cycle. IDH1 mutant and IDH1 wild-type exhibit distinct pathogenic profiles. The IDH1 mutation has become a standard diagnostic criterion for glioma classification, with numerous studies indicating its role as an independent prognostic factor in glioma patients. IDH1 mutation signifies a more favorable prognosis, and individuals possessing this mutation are more likely to get advantages from GTR [31,32]. Therefore, preoperative assessment of IDH1 status is valuable for surgical planning and prognostic prediction.

Our study demonstrated a correlation between IDH1 mutation status and glioma grade, with IDH mutations being more common in LGG. Regardless of histological grade, SMI-based tumor vascular architecture proved to be an effective predictor of IDH1 mutation status. In MRI, characteristics associated with tumor vasculature have been utilized to differentiate the molecular subtypes of gliomas. Microvascular caliber MRI may noninvasively evaluate microvascular attributes by quantifying microvascular diameter and density following the injection of contrast agents [33,34,35]. Parameters derived from microvascular caliber imaging show strong correlation with histologic findings, indicating that IDH1 mutant gliomas have fewer and less dense micro-vessels [35,36,37]. Furthermore, prevalent MRI cerebral perfusion imaging modalities, such as dynamic contrast enhancement, dynamic susceptibility contrast, and arterial spin labeling, have demonstrated that the blood volume and blood flow in IDH1 mutant gliomas are comparatively diminished, indicating reduced angiogenesis in these tumors [38,39]. Similarly, IDH1 mutant and wild-type tumors differ significantly in volume transfer constant (Ktrans), a commonly used DCE-MRI parameter, indicating differences in blood–brain barrier permeability based on IDH1 status [40]. IDH1 wild-type gliomas exhibited higher blood perfusion, and MRI sensitivity for diagnosing IDH1 mutations ranged between approximately 75.0% and 85.0% [41].

In our study, wild-type IDH1 gliomas exhibited a more malignant SMI blood flow pattern and pronounced peritumoral edema. Subsequent investigation revealed a distinct correlation between blood flow architecture and IDH1 expression. The logistic model achieved an accuracy of 81.3%, a sensitivity of 90.9%, and a specificity of 73.1% in classifying IDH1 expression. It possesses comparable diagnostic effectiveness to MRI. The blood flow signals in IDH1 wild-type gliomas are comparatively plentiful and significantly distorted, aligning with findings from prior MRI perfusion assessments. The IDH1 wild type glioma may result in elevated expression of hypoxia-inducible factor-1, subsequently increasing the expression of angiogenic factors in hypoxic tissues. This stimulation promotes the mitosis of vascular endothelial cells, leading to extensive immature neovascularization. Neovascularization is susceptible to arteriovenous fistula and vascular malformation, resulting in tortuosity and dilation of blood vessels inside the tumor and tortuosity of arteries around the tumor [38,42]. The IDH1 mutant type is more likely to exhibit a benign vascular architecture. Owing to the diminished proliferation and widespread infiltration, low-grade gliomas (LGGs) may exhibit a lesser degree of hypoxia compared to high-grade gliomas (HGGs), resulting in a comparatively reduced production of angiogenic factors. This further elucidates the relationship among vascular architecture, IDH1 expression, and glioma grade.

Subsequent examination of IDH1 expression across several histological grades indicated that in LGG, the Young’s modulus of the mutant variant surpassed that of the wild type. This aligns with the findings of Pepin et al. [40], who identified a distinct link between MRI elastography stiffness and IDH1 mutation status, indicating that IDH1 mutant tumors exhibit much more stiffness than IDH1 wild-type tumors. Conversely, Miroshnikova et al. [43] reached an opposing finding. Atomic force microscopy is a primary tool employed to assess hardness in laboratory settings, capable of identifying elastic mechanical characteristics at the nanoscale. A study on glioma nanomechanics revealed that IDH1 wild-type cells exhibit greater softness compared to IDH1 mutant cells, suggesting that mutants may influence tumor stiffness through the regulation of actin folding and binding, thereby mediating cytoskeletal reorganization, promoting cell sclerosis, and facilitating changes in cell morphology [44].

The macroscopic stiffness of a tumor is influenced by several variables, including cellular distribution and density, angiogenesis, collagen composition, etc. Alteration of any one element may influence the composition of the extracellular matrix, hence impacting the total elasticity value of the lesion. Our investigation revealed that soft tumor parenchyma correlated with wild-type IDH1 expression, and a highly malignant blood flow architecture was seen in all IDH1 wild-type gliomas, but IDH mutant gliomas exhibited a comparatively benign blood flow architecture. Although the difference lacked statistical significance, this implies that IDH1 expression may influence tumor angiogenesis. The microstructural mechanics and macroscopic tumor rigidity were subsequently altered. In LGG, the IDH1 mutant subtype has a greater incidence of malignant transformation. The stiffness value is anticipated to serve as an imaging biomarker for IDH1 expression levels in LGG to forecast malignant development; however, its predictive efficacy and the underlying pathophysiological mechanisms responsible for variations in stiffness require further study.

This study also has certain limitations, which may restrict the statistical power and general applicability of our research results. The limited cohort potentially reduces the ability to detect significant associations or subtle effects. Furthermore, the restricted sample may introduce selection bias and limit the external validity of the results, particularly in broader or more diverse populations. A further limitation of this study is that our analysis did not incorporate additional advanced imaging modalities, such as preoperative MR perfusion parameters or radiomic features. Conducting comparative analysis with such technologies will help to define the complementary role and specific clinical scenarios where each technique provides maximal diagnostic benefit more clearly. In this study, we conducted a qualitative assessment of the SMI vascular architecture, which is of particular significance. We acknowledge that while this shows good agreement in a controlled research setting, generalizability to broader clinical practice across multiple operators and centers may require further validation. Therefore, we emphasized the need for future multi-center studies, along with the adoption of standardized training and protocols, in order to further reduce the differences among operators, verify these initial observations, and enhance the reliability and applicability of the conclusions, which would help better define the complementary role and specific clinical scenarios where each technique provides maximal diagnostic benefit.

## Figures and Tables

**Table 1:** Clinical characteristics of patients.

	HGG	LGG	*p* Value
Age	55.6 ± 14.6	43.6 ± 12.4	0.004 *
Sex (*n*, %)			0.762
Female	12 (25.0%)	8 (16.3%)	
Male	18 (37.5%)	10 (20.8%)	
Tumor location (*n*, %)			1.000
Frontal	17 (35.4)	9 (18.8%)	
Temporal	4 (8.3%)	5 (10.4%)	
Parietal	2 (4.2%)	1 (2.1%)	
Frontoparietal	1 (2.1%)	2 (4.2%)	
Frontotemporal	2 (4.2%)	1 (2.1%)	
Occipital	1 (2.1%)	0 (0.0%)	
Parietooccipital	2 (4.2%)	0 (0.0%)	
Paracele	1 (2.1%)	2 (4.2%)	
Total (*n*, %)	30 (62.5%)	18 (37.5%)	

* *p* < 0.05 was considered statistically significant.

**Table 2:** Grayscale ultrasound characteristics of HGG and LGG.

	HGG (*n*, %)	LGG (*n*, %)	*p* Value
Morphology			0.632
Regular	10 (20.8%)	4 (8.3%)	
Irregular	20 (41.7%)	14 (29.2%)	
Boundary			0.178
Clear	14 (29.2%)	12 (25.0%)	
Unclear	16 (33.3%)	6 (12.5%)	
Depth			0.751
≤2 cm	26 (54.2%)	15 (31.3%)	
>2 cm	4 (8.3%)	3 (6.3%)	
Tumor size			0.940
≤5 cm	17 (35.4%)	10 (20.8%)	
>5 cm	13 (27.1%)	8 (16.7%)	
Peritumoral edema			0.048 *
None	4 (8.3%)	8 (16.7%)	
≤2 cm	13 (27.1%)	6 (12.5%)	
>2 cm	13 (27.1%)	4 (8.3%)	
Cystic change			0.084
Available	16 (33.3%)	5 (10.4%)	
None	14 (29.2%)	13 (27.1%)	
Calcification			0.105
Available	3 (6.3%)	6 (12.5%)	
None	27 (56.3%)	12 (25.0%)	
Total	30 (62.5%)	18 (37.5%)	

* *p* < 0.05 was considered statistically significant.

**Table 3:** SMI evaluation of vascular architecture in tumor and peritumoral tissues.

	HGG (*n*, %)	LGG (*n*, %)	*p* Value
Tumor Vessels			0.001 *
Dilated and bent vessels	18 (37.5%)	2 (4.2%)	
Straight and branching vessels	7 (14.6%)	13 (27.1%)	
Avascular	5 (10.4%)	3 (6.3%)	
Vessels Around the Tumor			0.001 *
Distorted and surrounding vessels	20 (41.7%)	2 (4.2%)	
Straight and penetrating vessels	6 (12.5%)	9 (18.8%)	
Normal cerebral vessels	4 (8.3%)	7 (14.6%)	
Total	30 (62.5%)	18 (37.5%)	

* *p* < 0.05 was considered statistically significant.

**Table 4:** Young’s modulus values of intratumoral and peritumoral tissues.

	LGG (kPa)	HGG (kPa)	t’	*p* Value
Intratumoral tissue	23.4 ± 11.6	12.1 ± 13.7	2.937	0.005 *
Peritumoral tissue	13.2 ± 4.6	10.4 ± 3.6	2.305	0.026 *
t’	3.499	0.644		
*p* value	0.001 *	0.524		

* *p* < 0.05 was considered statistically significant.

**Table 5:** Correlation between IDH1 expression and ultrasound characteristics in gliomas.

	Mutant Type (*n*, %)	Wild Type (*n*, %)	*p* Value
Morphology			0.654
Regular	9 (18.8%)	9 (18.8%)	
Irregular	13 (27.1%)	17 (35.4%)	
Boundary			0.516
Clear	16 (33.3%)	21 (43.8%)	
Unclear	6 (12.5%)	5 (10.4%)	
Depth			0.687
≤2 cm	18 (37.5%)	23 (47.9%)	
>2 cm	4 (8.3%)	3 (6.3%)	
Tumor size			0.422
≤5 cm	11 (22.9%)	16 (33.3%)	
>5 cm	11 (22.9%)	10 (20.8%)	
Peritumoral edema			0.036 *
None	11 (22.9%)	6 (12.5%)	
≤2 cm	9 (18.8%)	10 (20.8%)	
>2 cm	2 (4.2%)	10 (20.8%)	
Cystic change			0.715
Available	9 (18.8%)	12 (25.0%)	
None	13 (27.1%)	14 (29.2%)	
Calcification			0.181
Available	8 (16.7%)	4 (8.3%)	
None	14 (29.2%)	22 (45.8%)	
Tumor Vessels			0.002 *
Dilated and bent vessels	3 (6.3%)	17 (35.4%)	
Straight and branching vessels	13 (27.1%)	7 (14.6%)	
Avascular	6 (12.5%)	2 (4.2%)	
Vessels Around the Tumor			0.001 *
Distorted and surrounding vessels	4 (8.3%)	18 (37.5%)	
Straight and penetrating vessels	12 (25.0%)	3 (6.3%)	
Normal cerebral vessels	6 (12.5%)	5 (10.4%)	
Intratumoral Young’s modulus	17.8 ± 10.3	15.1 ± 16.6	0.514
Total (*n*, %)	22 (45.8%)	26 (54.2%)	

* *p* < 0.05 was considered statistically significant.

**Table 6:** Logistic regression analysis of IDH1 expression in gliomas.

	OR (95%CI)	*p* Value
Tumor Vessels		
Straight and branching vessels and Avascular	1.00	
Dilated and bent vessels	0.118 (0.020–0.682)	0.017 *
Vessels Around the Tumor		
Straight and penetrating vessels and Normal cerebral vessels	1.00	
Distorted and surrounding vessels	0.123 (0.018–0.828)	0.031 *
Peritumoral edema		
None	1.00	
≤2 cm	3.061 (0.381–24.601)	0.293
>2 cm	0.314 (0.035–2.784)	0.298

* *p* < 0.05 was considered statistically significant.

**Table 7:** Correlation between IDH1 expression and ultrasound characteristics in LGG and HGG.

	LGG		*p* Value	HGG		*p* Value
	Mutant Type	Wild Type		Mutant Type	Wild Type	
Morphology			1.000			0.236
Regular	3 (6.3%)	1 (2.1%)		6 (12.5%)	8 (16.7%)	
Irregular	10 (20.8%)	4 (8.3%)		3 (6.3%)	13 (27.1%)	
Boundary			1.00			0.694
Clear	9 (18.8%)	3 (6.3%)		5 (10.4%)	9 (18.8%)	
Unclear	4 (8.3%)	2 (4.2%)		4 (8.3%)	12 (25.0%)	
Depth			1.000			1.000
≤2 cm	11 (22.9%)	4 (8.3%)		7 (14.6%)	19 (39.6%)	
>2 cm	2 (4.2%)	1 (2.1%)		2 (4.2%)	2 (4.2%)	
Tumor size			0.314			1.000
≤5 cm	6 (12.5%)	4 (8.3%)		5 (10.4%)	12 (25.0%)	
>5 cm	7 (14.6%)	1 (2.1%)		4 (8.3%)	9 (18.8%)	
Peritumoral edema			1.000			0.498
None	10 (20.8%)	3 (6.3%)		1 (2.1%)	3 (6.3%)	
≤2 cm	3 (6.3%)	2 (4.2%)		6 (12.5%)	8 (16.7%)	
>2 cm	0 (0.0%)	0 (0.0%)		2 (4.2%)	9 (18.8%)	
Cystic change			0.522			0.704
Available	3 (6.3%)	0 (0.0%)		6 (12.5%)	12	
None	10 (20.8%)	5 (10.4%)		3 (6.3%)	9 (18.8%)	
Calcification			1.000			0.207
Available	6 (12.5%)	3 (6.3%)		2 (4.2%)	1 (2.1%)	
None	7 (14.6%)	2 (4.2%)		7 (14.6%)	20 (41.7%)	
Tumor Vessels			0.109			0.109
Dilated and bent vessels	0 (0.0%)	2 (4.2%)		3 (6.3%)	15 (31.3%)	
Straight and branching vessels	10 (20.8%)	2 (4.2%)		3 (6.3%)	4 (8.3%)	
Avascular	3 (6.3%)	1 (2.1%)		3 (6.3%)	2 (4.2%)	
Vessels Around the Tumor			0.061			0.103
Distorted and surrounding vessels	0 (0.0%)	2 (4.2%)		4 (8.3%)	16 (33.3%)	
Straight and penetrating vessels	8 (16.7%)	1 (2.1%)		4 (8.3%)	2 (4.2%)	
Normal cerebral vessels	5 (10.4%)	2 (4.2%)		1 (2.1%)	3 (6.3%)	
Intratumoral elasticity values of SWE	27.0 ± 11.3	14.2 ± 6.5	0.031 *	12.5 ± 9.2	11.9 ± 15.5	0.904
Total (*n*, %)	13 (27.1%)	5 (10.4%)		9 (18.8%)	21 (43.8%)	

* *p* < 0.05 was considered statistically significant.

## Data Availability

The original contributions presented in this study are included in the article. Further inquiries can be directed to the corresponding authors.
